# Knowledge gaps and research priorities in tuberculous meningitis

**DOI:** 10.12688/wellcomeopenres.15573.1

**Published:** 2019-11-28

**Authors:** James A Seddon, Robert Wilkinson, Reinout van Crevel, Anthony Figaji, Guy E Thwaites, Tuberculous Meningitis International Research Consortium, Rob E. Aarnoutse, Rob E. Aarnoutse, Suzanne T. B. Anderson, Suzanne T. B. Anderson, Nathan C. Bahr, Nguyen D. Bang, David R. Boulware, Tom Boyles, Lindsey H. M. te Brake, Satish Chandra, Felicia C. Chow, Fiona V. Cresswell, Reinout van Crevel, Angharad G. Davis, Sofiati Dian, Joseph Donovan, Kelly E. Dooley, Anthony Figaji, A. Rizal Ganiem, Ravindra Kumar Garg, Diana M. Gibb, Raph L. Hamers, Nguyen T. T. Hiep, Darma Imran, Akhmad Imron, Sanjay K. Jain, Sunil K. Jain, Byramee Jeejeebhoy, Jayantee Kalita, Rashmi Kumar, Vinod Kumar, Arjan van Laarhoven, Rachel P-J. Lai, Abi Manesh, Suzaan Marais, Vidya Mave, Graeme Meintjes, David B. Meya, Usha K. Misra, Manish Modi, Alvaro A. Ordonez, Nguyen H. Phu, Sunil Pradhan, Kameshwar Prasad, Alize M. Proust, Lalita Ramakrishnan, Ursula Rohlwink, Rovina Ruslami, Johannes F. Schoeman, James A. Seddon, Kusum Sharma, Omar Siddiqi, Regan S. Solomons, Nguyen T. T. Thuong, Guy E. Thwaites, Ronald van Toorn, Elizabeth W. Tucker, Sean A. Wasserman, Robert J. Wilkinson

**Affiliations:** 1 Infectious Diseases, Imperial College London, London, W2 1PG, UK; 2 Paediatric Infectious Diseases, Imperial College Healthcare NHS Trust, St. Mary's Campus, London, W2 1PG, UK; 3 Desmond Tutu TB Centre, Department of Paediatrics and Child Health, Stellenbosch University, Cape Town, Western Cape, 8005, South Africa; 4 Wellcome Centre for Infectious Diseases Research in Africa, Institute of Infectious Disease and Molecular Medicine and Department of Medicine, University of Cape Town, Observatory, 7925, South Africa; 5 Francis Crick Institute, London, NW1 1AT, UK; 6 Department of Internal Medicine and Radboud Center for Infectious Diseases, Radboud University Nijmegen Medical Centre, Nijmegen, The Netherlands; 7 Centre for Tropical Medicine and Global Health, Nuffield Department of Medicine, University of Oxford, Oxford, UK; 8 Neuroscience Institute, Division of Neurosurgery, University of Cape Town, Cape Town, South Africa; 9 Oxford University Clinical Research Unit, University of Oxford, Ho Chi Minh City, Vietnam

**Keywords:** Tuberculosis, Tuberculous Meningitis, Research Priorities, Epidemiology, Diagnosis, Treatment, Critical Care, Care Cascade

## Abstract

Tuberculous meningitis (TBM) is the most severe and disabling form of tuberculosis (TB), accounting for around 1-5% of the global TB caseload, with mortality of approximately 20% in children and up to 60% in persons co-infected with human immunodeficiency virus even in those treated. Relatively few centres of excellence in TBM research exist and the field would therefore benefit from greater co-ordination, advocacy, collaboration and early data sharing. To this end, in 2009, 2015 and 2019 we convened the TBM International Research Consortium, bringing together approximately 50 researchers from five continents. The most recent meeting took place on 1 ^st^ and 2 ^nd^ March 2019 in Lucknow, India. During the meeting, researchers and clinicians presented updates in their areas of expertise, and additionally presented on the knowledge gaps and research priorities in that field. Discussion during the meeting was followed by the development, by a core writing group, of a synthesis of knowledge gaps and research priorities within seven domains, namely epidemiology, pathogenesis, diagnosis, antimicrobial therapy, host-directed therapy, critical care and implementation science. These were circulated to the whole consortium for written input and feedback. Further cycles of discussion between the writing group took place to arrive at a consensus series of priorities. This article summarises the consensus reached by the consortium concerning the unmet needs and priorities for future research for this neglected and often fatal disease.

## Introduction

Tuberculous meningitis (TBM) is caused when *Mycobacterium tuberculosis* enters the cerebrospinal fluid (CSF), leading to inflammation of the meninges, and subsequent clinical features of meningitis ^1^. Cerebral ischaemia, hydrocephalus, raised intracranial pressure, and cranial nerve dysfunction commonly occur. TBM is the most severe form of tuberculosis (TB) and is universally fatal if untreated. Even with treatment, mortality is high, and some degree of morbidity is near universal. Our current understanding of the epidemiology, natural history and pathophysiology of TBM is limited and it will only be through an improved appreciation of these areas that appropriate clinical and public health interventions will be possible. Current diagnostic tools have inadequate test performance, meaning that most individuals with TBM are either not diagnosed or diagnosed late, once significant cerebral damage has occurred. Current antimicrobial treatment is based mainly on the treatment of pulmonary TB with little consideration to mode of drug action or CSF penetration. Given that most damage caused by TBM is due to host inflammation, it is anticipated that host-directed therapies should play an important role in modulating the damaging immune-medicated effects. However, other than corticosteroids, no agent has been demonstrated to be effective in reducing mortality and corticosteroids have not been shown to improve long-term morbidity ^2^. The critical care management of TBM is also poorly understood, with highly divergent practice in the management of raised intracranial pressure, hydrocephalus, blood pressure, oxygenation and hyponatraemia ^3^. Understanding the optimal management of these areas would almost certainly improve the long-term outcome of diagnosed patients. Finally, the management of TBM within health systems is sub-optimal with services and tools rarely provided at the locations where patients present. Public sensitisation is infrequently undertaken, and clinical training is often limited.

Globally, there are relatively few researchers of TBM and only a small number of centres of excellence. The field, therefore, would benefit from greater co-ordination, collaboration and data sharing. In 2009, the TBM International Research Consortium was formed and met for the first time in Cape Town, South Africa. Subsequent meetings took place in 2015 in Dalat, Vietnam, and in 2019 in Lucknow, India. The Consortium brings together approximately 50 experts from five continents. At the Lucknow meeting, the Consortium felt that it would be useful to develop a document detailing research priorities in TBM. The practice of establishing knowledge gaps and priorities for research helps researchers to focus efforts on areas that are perceived to be most likely to lead to clinical benefit. It also allows funders to target their funding. This practice can also be beneficial for advocacy through a clearer message and co-ordinated agenda. In this article we describe the process of developing a TBM research agenda and outline the priorities that were developed.

## Identifying knowledge gaps and research priorities for TBM

The 3 ^rd^ TBM International Research Consortium took place in Lucknow on the 1 ^st^ and 2 ^nd^ March 2019. During the meeting, research updates were presented by leading researchers and clinicians in each respective field. Each had been requested to additionally present their thoughts and suggestions for knowledge gaps and research priorities in that field. Following each presentation, debate around these gaps and priorities took place. After the meeting, these priorities were assimilated into tables of research gaps by a core writing group, with gaps grouped into the themes of epidemiology, pathogenesis, diagnosis, antimicrobial therapy, host-directed therapy, critical care and care cascades. For each knowledge gap, study designs were proposed that might address them. These tables were circulated to the whole consortium who were asked to provide input and make suggestions as to which areas were the highest priority. Through further cycles of discussion within the writing group, the tables of knowledge gaps were updated ( Table 1– Table 7) and a consolidated list of top priorities for TBM research was developed, which are presented in Table 8. This article describes the knowledge gaps and research priorities for TBM.

**Table 1.** Knowledge gaps in the epidemiology of tuberculous meningitis.

	**Question**	**Potential study designs**
**Understanding burden**	How many children/adults in each country develop TBM each year?	• Systematic review of the pre-chemotherapy literature to identify proportion of TB cohorts that are TBM/which patients get TBM • Cross-sectional studies/analysis of TB registers to identify what proportion of TB cohorts are TBM/which patients get TBM • Modelling studies to quantify burden
	How many children/adults die from TBM in each country each year?	• Autopsy studies of deaths from clinical meningitis • Systematic reviews of published studies that report on outcome of cohorts of TBM • Analysis of register data at district, national and international level to evaluate proportion of outcomes for TBM that are registered as death • Modelling studies to evaluate the number of deaths from TBM, given number of cases, the proportion diagnosed and expected mortality treated and untreated
	What is the morbidity from TBM?	• Systematic reviews of published studies that have evaluated the neurological disability in survivors of TBM • Modelling studies to quantify the burden of morbidity in different countries and regions given the burden of treated TBM • Cohorts of children and adults with long-term neurocognitive outcomes and quality of life documented
	What is the cost of TBM?	• Collection of costing data in different settings to determine the cost of diagnosis and treatment of TBM, as well as the cost of caring for an individual who is disabled by TBM from a health system and societal perspective • Health economic evaluations to determine the health system and family costs of TBM diagnosis, treatment, mortality and disability in terms of life years, DALYs and QALYs lost due to TBM
**Individual risk factors for** **developing TBM**	Which adults and children are most likely to develop TBM following TB exposure and infection?	• Systematic reviews of household contact studies to identify risk factors in household contacts of developing both TB and TBM • Cohort studies of individuals who have been exposed to TB and followed to determine who progresses to TBM and evaluates risk factors for progression • Case control studies of people who had been exposed to TB who developed TBM, compared to TB-exposed individuals who developed pulmonary TB or no TB. • Cross-sectional study comparing TBM cases with pulmonary TB cases, to evaluate which patients with TB are at increased risk of TBM
	Which adults and children are at high risk of developing drug- resistant TBM?	• Evaluation of treatment resisters to compare cases of drug- resistant TBM with drug-susceptible TBM • Cross-sectional studies of TBM cases, comparing those with drug- resistant TBM with those with drug-susceptible TBM
**Understanding risk** **factors for outcome in** **TBM**	Which adults and children are at risk of dying once diagnosed with TBM?	• Evaluation of treatment registers to identify risk factors for mortality among TBM cases • Cohort studies of TBM patients with evaluation of risk factors for mortality • Biomarker research to determine if any samples taken at baseline or during treatment predict mortality
	Which clinical and biological characteristics are associated with poor outcome?	• Cohort studies of TBM patients with evaluation of baseline and follow up risk factors for mortality and morbidity
	Which adults and children are at risk of developing severe neurological disability if the survive TBM?	• Cohort studies of TBM patients to identify risk factors for morbidity in survivors • Biomarker research to determine if any samples taken at baseline or during treatment predict morbidity in survivors

TBM, tuberculous meningitis; DALY, disability adjusted life year; QALY, quality adjusted life year; TB, tuberculosis.

**Table 2.** Knowledge gaps in our understanding of the pathogenesis of tuberculous meningitis.

	Question	Potential study designs (see legend)
**CNS penetration**	How do bacilli cross the intact blood brain barrier?	*A, B*
	How does Bacille Calmette Guérin confer protection against TBM?	*B, C*
**CNS inflammation**	What are the soluble and cellular determinants of brain inflammation?	*A, B, D*
	What are the modifying effects of age, host genotype and HIV co-infection on these responses?	D
**Metabolic derangement**	What patterns of metabolic derangement does CNS inflammation establish?	*A, B, D*
	How does metabolic derangement contribute to cerebral dysfunction and brain injury?	*A, D*
**Mycobacterial characteristics**	How do different strain types of *M tuberculosis* impact on disease pathogenesis?	*A, D*
**Ongoing brain injury**	What patterns of brain injury contribute to poor outcome in TBM?	*A, B, D*
	By what mechanisms is brain injury perpetuated in TBM?	*A, B, D*
**Advanced and dynamic** **cerebral imaging studies**	Which areas of the brain are most affected by TBM?	B, imaging
	Which areas and patterns of brain damage correlate with poor outcome or good resolution of TBM	*B, D*

TBM, tuberculous meningitis; CNS, central nervous system; HIV, human immunodeficiency virus. A. *In vitro* models including isolated cell populations, blood brain barrier models and cerebral organoids. B. *In vivo* models including zebrafish, mice, rabbits and non-human primates. C. Correlates of protection studies in humans. D. Observational, diagnostic and randomised intervention studies in humans.

**Table 3.** Knowledge gaps in the diagnosis of tuberculous meningitis.

	Question	Potential study designs
**Pathogen-** **based**	Do novel nucleic amplification tests improve sensitivity, management and outcome of TBM?	• Adequately designed and powered STARD compliant multicentre diagnostic evaluations that encompass important subgroups including children, HIV co-infected persons and those with drug resistant disease. • Biobanking of well-characterised samples to aid discovery and evaluation of novel diagnostic modalities
	Do novel tests that rely on *M. tuberculosis* derived molecules or metabolites have useful rule-in value?	
	Is metagenomic sequencing a feasible and accurate means to detect *M. tuberculosis* in CSF?	
**Host-response** **based**	Can single or multiplexed combinations of host- derived molecules adequately rule out TBM when applied to CSF, serum of peripheral blood?	• Capitalise on interest in the development of point of care tests for all forms of tuberculosis and specifically apply to TBM • Adequately designed and powered STARD compliant multicentre diagnostic evaluations that encompass important subgroups including children, HIV co-infected persons and those with drug resistant disease. • Biobanking of well-characterised samples to aid discovery and evaluation of novel diagnostic modalities
**Clinical** **algorithms**	Do clinical algorithms have useful diagnostic accuracy when evaluated against microbiological and composite endpoints?	• Adequately designed and powered STARD compliant multicentre diagnostic evaluations that encompass important subgroups including children, HIV co-infected persons and those with drug-resistant disease.
	Do clinical algorithms positively or negatively influence patient outcome?	
**Diagnostic** **Strategies**	How can clinical, pathogen and host-response diagnostic approaches be combined into a diagnostic strategy?	• Integrated Phase 1, 2 and 3 studies to evaluate how different individual tests might be combined to diagnose TBM

CSF, cerebrospinal fluid; TBM, tuberculous meningitis; STARD, Standards for Reporting Diagnostic Accuracy; HIV, human immunodeficiency virus.

**Table 4.** Knowledge gaps in the development of anti-microbial drugs for tuberculous meningitis and our understanding drug distribution.

	Question	Potential study designs (see legend)
**Higher dose antibiotics**	Does high-dose rifampicin improve outcome?	D
	Should higher-dose isoniazid be prescribed to rapid acetylators or to all TBM patients?	D
**Alternative drugs**	Can linezolid or other 2 ^nd^ line drugs improve outcome?	B, D
**Better drug delivery**	Can modification of drug delivery (e.g. by liposome or nanoparticle-mediated permeation) improve outcome?	A, B, D
	Does inhibition of efflux mechanisms improve outcome?	A, B, D
**Optimal treatment in specific** **patient categories**	How can we improve outcomes in HIV-infected patients, children, and individuals with drug-resistant TBM?	C, D
	What is the optimal duration and regimen for tuberculomas and spinal TB?	D
**Study design**	What is the best way to find optimal treatment regimens for TBM?	B, D

TBM, tuberculous meningitis; HIV, human immunodeficiency virus; TB, tuberculosis. A. *In vitro* models including isolated cell populations, blood brain barrier models and cerebral organoids. B. *In vivo* models including zebrafish, mice, rabbits and non-human primates. C. Correlates of protection studies in humans. D. Observational, diagnostic and randomised intervention studies in humans.

**Table 5.** Knowledge gaps in the understanding and use of host directed therapies for tuberculous meningitis.

	Question	Potential study designs
**Corticosteroids**	What is the best drug, dose, route of administration and duration?	• Comparative randomised trials against the current standard of dexamethasone in adults, and prednisolone in children
	What determines corticosteroid treatment effect and how do they improve outcomes?	• Unbiased characterisation of the cerebral pathophysiology of TBM, using serial brain imaging and CSF analysis of inflammatory and metabolic pathways, in those treated with or without corticosteroids (ideally linked to a randomised comparison)
	Do corticosteroids prevent or reduce the severity of cerebral immune reconstitution inflammatory syndrome in patients co- infected with HIV and starting anti-retroviral therapy	• Randomised placebo-controlled trial of corticosteroids given at the initiation of antiretroviral therapy
**Thalidomide**	What is the role of thalidomide in the management of TBM?	• Randomised trial of thalidomide for corticosteroid- refractory complications of TBM (e.g. tuberculomas, vasculitis with multiple infarcts)
**Anti-TNF biological agents** **(e.g. infliximab)**	Do they have a role in the management of the inflammatory complications of TBM?	• *In vivo* models, including rabbits and mice • Randomised trial of anti-TNF biologic, possibly versus thalidomide, for the treatment of corticosteroid refractory tuberculomas and vasculitis with multiple infarcts
**Aspirin**	Does aspirin reduce death and disability from TBM?	• Phase 3 randomised controlled trials of aspirin in adults and children
	How does aspirin influence pathophysiology and improve outcomes	• *In vivo* models, including rabbits and mice • Systematic characterisation of brain imaging and CSF inflammatory (including lipid-mediated) and metabolic pathways in those recruited to aspirin trials
**Customised host directed** **therapies, targeting molecules** **and/or pathways known to** **influence pathophysiology and** **clinical outcome of TBM**	How can new targets for host directed therapy be identified?	• *In vivo* models, including rabbits and mice • Unbiased ‘omics’ approach to investigate genes, proteins, inflammatory mediators and metabolites that associate with clinical endpoints, including inflammatory complications occurring after the start of treatment and longer-term survival and disability
	Are there drugs widely and safely used in the treatment of other diseases that can be re-purposed for host directed therapy for TBM? For example, verapamil, doxycycline and metformin	• *In vivo* models, including rabbits and mice • Phase 2 clinical trials exploring dose, safety, and potential efficacy. Require details linked laboratory and imaging studies to support potential clinical efficacy with mechanistic understanding
**Personalized use of host-** **directed therapy**	Can use of simple biomarkers or genetic traits help personalize choice of adjuvant treatment?	• Randomised controlled trial stratifying steroid-use by LTA4H-genotype • Mendelian randomization or stratification of results of randomised controlled trials examining host-directed therapy by blood or CSF biomarker profiles
**Optimal treatment in specific** **patient categories**	HIV-infected patients, children, MDR- and XDR-TB disease	• Sub-group analysis in randomised controlled trials • Specific randomised controlled trials

TBM, tuberculous meningitis; TNF, tumour necrosis; CSF, cerebrospinal fluid; LTA4H, Leukotriene-A4 hydrolase, MDR, multidrug-resistant; XDR, extensively drug-resistant; TB, tuberculosis; HIV, human immunodeficiency virus.

**Table 6.** Knowledge gaps in our understanding and use of critical care in tuberculous meningitis.

	**Question**	**Potential study designs**
**Acute management of** **hydrocephalus**	How should raised intracranial pressure be managed in the acute setting?	• Trials of medical therapy, continuous lumbar drainage, external ventricular drainage, early ventriculoperitoneal shunting/ETV
**Subacute/Long** **term management of** **hydrocephalus**	Should hydrocephalus be managed based on communicating versus non-communicating level of CSF block?	• Trial of surgical (based on agreed criteria) versus medical therapy first in patients with communicating hydrocephalus
	For surgically treated patients, is ventriculoperitoneal shunting or endoscopic third ventriculostomy preferable?	• Where surgery is indicated, trial of shunting versus endoscopy
**Treatment thresholds for** **raised intracranial pressure**	Should treatment thresholds for raised intracranial pressure be based on standard norms or should it be more aggressive?	• Compare standard versus more aggressive threshold for ICP treatment
**Monitoring of intracranial** **pressure**	Is intracranial pressure monitoring valuable?	• Compare standard of care (where ICP is not monitored) with treatment directed by monitored ICP
	What technique is optimal, considering availability, safety, effectiveness, and economics?	• Compare current options for monitoring intracranial pressure clinically
	What non-invasive techniques are valuable in screening for and/or monitoring raised intracranial pressure?	• Compare current (and novel) methods of non- invasive ICP measurement with measured ICP in a real-world setting
**Blood pressure** **management**	Is there benefit from blood pressure optimisation/ blood pressure augmentation?	• Compare standard of care with targeted blood pressure management
	If cerebral perfusion pressure monitoring is possible, what should the target be, normal or supra-normal?	• Compare minimum thresholds of CPP (based on current recommendations) with CPP augmentation
	What methods of blood pressure management are preferable?	• After basic resuscitation, compare intravascular fluids with inotropes, particularly with vasopressors
**Systemic oxygenation**	Does supplemental oxygen improve brain oxygenation?	• Compare standard of care, supplemental oxygen, and normobaric hyperoxia
**Hyponatraemia**	Does the treatment of hyponatremia depend on the correct diagnosis?	• Compare standard therapy for all patients (saline/ hypertonic saline) versus (attempted) diagnosis- based directed therapy
	What is the safest and most effective protocol for treating hyponatremia?	• Compare saline therapy, hypertonic saline, fludrocortisone
**Early prognostic indicators** **to direct acute therapy**	• What are the early clinical and radiological early warning signs for patients at high risk of deterioration and outcome?	• Develop a multivariable predictive score
	Are there criteria for futility of care?	• Determine a predictive threshold of the score for futility of care

CSF, cerebrospinal fluid; ETV, endoscopic third ventriculostomy; ICP, intracranial pressure; CPP, cerebral perfusion pressure.

**Table 7.** Knowledge gaps related to the cascade of care for tuberculous meningitis ^33^.

	Question	Potential study designs
**Cascade** **of Care**	What are barriers to accessing appropriate services for TBM?	• Patient pathway analyses • Interview and focus groups of TBM patients, families and community health professionals
	What are barriers to adequate and timely clinical TBM diagnosis, including performing lumbar puncture?	• Interview and focus groups of TBM patients, families and community health professionals • Establishment and measurement of quality indicators for diagnosis
	What are barriers to improve quality of care for TBM in hospital and increase the proportion of patients discharged alive?	• Recording of in-hospital mortality • Interview and focus groups of doctors and nurses involved in TBM care • Establishment and measurement of quality indicators for in- hospital care
	What are barriers to retain patients to care after hospital discharge?	• Recording of loss to follow-up after hospital discharge • Interview and focus groups of patients, families and health providers in ambulatory care • Establishment and measurement of quality indicators for post- discharge care
	What are barriers to provide necessary care (e.g. rehabilitation) after hospital discharge?	• Establishment of indications for additional care and measurement of care provided • Interview and focus groups of patients, families and health providers in ambulatory care
**Health** **systems** **factors**	What is the availability of facilities with necessary high-level care for TBM and neurologists trained in TBM?	• Health systems research; assessment of available health statistics, facilities, interviews with health providers etc
	How can incidence and outcome data be collected and reported?	• Collection of TBM cohort data • Interviews with health providers, national programs etc
	What national protocols and patient management protocols are available?	• Interviews with health providers, national programs etc
	How does the health system organisation and regulation affect TBM care (e.g. in terms of referral systems)?	• Health systems research; assessment of available health statistics, facilities, interviews with policy makers, health providers etc
	What are financial barriers to TBM care? how is TBM care covered in public/private health insurance?	• Health-economic studies including patient / family interviews
	What health information should be given to patients and health professionals?	• Knowledge assessment; establishment of minimum requirement of knowledge for patients and health professionals

TBM, tuberculous meningitis.

**Table 8.** Research priorities in tuberculous meningitis.

Theme	Research priorities
**Epidemiology**	• To identify individual characteristics associated with poor outcome in TBM • To quantify the burden and outcome of TBM across different sites and in different risk groups
**Pathogenesis**	• To identify the causes brain injury using pathway analysis in order to determine which pathways should be targeted • To determine the pattern of brain injury using advanced and dynamic cerebral imaging
**Diagnosis**	• To evaluate strategies that use currently-available tools (such as Xpert Ultra) in rigorous multicentre studies to determine sensitivity and specificity across relevant sub-groups and in different contexts • To evaluate host-based biomarkers of CSF that do not rely on mycobacterial identification for the diagnosis of TBM • To evaluate non-CSF tests for the diagnosis of TBM
**Anti-Microbial Drug** **Therapy**	• To determine if higher doses of rifampicin improve outcome in adults and children with TBM • To evaluate if the inclusion of newer drugs, such as linezolid, improve outcome • To evaluate novel mechanisms of drug delivery to the sites of disease
**Host directed therapy**	• To identify which patients will benefit most from corticosteroids and which will not • To determine how to manage steroid-resistant paradoxical reactions • To determine if aspirin improves outcome
**Critical Care**	• To evaluate the optimal method for managing raised intracranial pressure • To quantify the target systemic blood pressure for minimal brain injury evolution in TBM • To identify the optimal method of treating hyponatraemia • To determine the minimum package of supportive care that can improve outcome in TBM that can be delivered in a low-resource setting
**Cascade of Care**	• To establish the cascade of care for TBM patients in different settings • To identify factors associated with loss or delay across the cascade in different settings • To develop and test interventions aimed at improving the cascade of care for TBM in different settings

TBM, tuberculous meningitis; CSF, cerebrospinal fluid.

## Epidemiology

Accurate estimates of the incidence of TBM are lacking, in part as many cases die prior to diagnosis. Adult autopsy studies demonstrate that a high proportion of those with human immunodeficiency virus (HIV), in a high TB-burden setting, die from undiagnosed disseminated TB ^4^. Given that *M. tuberculosis* is now the most common cause of bacterial meningitis in some high TB burden settings ^5^, following wide implementation of routine vaccinations to meningococcus, pneumococcus and haemophilus, it is likely that this situation is replicated in children.

The World Health Organization (WHO) estimates that 10 million individuals developed TB in 2017 ^6^. Of the 6.4 million cases that were notified to WHO that year, 14% were classified as extrapulmonary ^6^. Given that a patient with any pulmonary involvement is classified by WHO as a pulmonary case (such as an individual with both pulmonary TB and TBM) ^7^, the proportion of patients who have extrapulmonary involvement is higher than this. To be notified, an individual must have been diagnosed and so it is likely that TBM is under-represented in cases notified to WHO.

Data on the proportion of all TB cases that have extrapulmonary involvement is limited and likely varies with multiple factors including the age and gender characteristics of the population, the HIV prevalence, where the cohort is recruited from (hospital vs. community), and the force of TB infection. The proportion of all TB patients that have TBM is even less unclear. A population-based estimate in a low TB-prevalence setting suggested that around 1% of TB cases were TBM ^8^, while a paediatric hospital-based cohort of confirmed TB in a high TB-burden setting suggested that over 10% of TB cases were TBM ^9^. If 1% of the overall TB burden is TBM then 100,000 individuals develop TBM each year. This figure could be significantly higher. TBM is universally fatal if untreated ^10^ but estimates of mortality in treated TBM are poor. A fifth of children die, with half of survivors experiencing neurodisability ^11^. In some cohorts of HIV-infected adults, mortality is as high as 60% ^12, 13^. To improve estimates, it would be informative to review the extensive pre-chemotherapy literature to both quantify the proportion of meningitis deaths that are caused by *M. tuberculosis* as well as to define what proportion of all TB cases are TBM and how this varies by age. It would also be useful to carry out contemporary studies to document the proportion of TB cases that are TBM in large cohorts of routine data or research cohorts, and document outcomes in those treated. Mathematical modelling could be used to estimate the incidence of TBM, as well as mortality.

Improved estimates of incidence and mortality would be vital for appropriate advocacy and resource planning. It would also be important to quantify morbidity in TBM survivors and the cost impact of TBM to society. TBM commonly causes profound neurodisability in survivors, requiring ongoing medical care and extensive community/family support. The degree of long-term disability in survivors has been poorly quantified, especially the impact on education, employment and quality of life. Better estimates of morbidity and cost to society could be developed through collection of appropriate qualitative, neurodevelopmental and costing data, combined with modelling studies.

Beyond burden estimates, it would be useful to better understand why some individuals develop TBM and characteristics that influence clinical outcome. These could be elucidated through systematic review of published studies, as well as larger, prospective clinical cohorts. Knowledge gaps for TBM epidemiology are shown in Table 1.

## Pathogenesis

The pathogenesis of TBM has recently been reviewed in depth ^14^. The classic paradigm is that meningitis arises as a consequence of bloodborne dissemination, with a single breach of the blood brain barrier being sufficient to form a central nervous system (CNS) focus ^15^, although this has been challenged ^16^. Whether bacilli predominantly transit the endothelium directly or within infected cells is incompletely understood, with a recent study suggesting both are possible ^17^. This work was performed using *Mycobacterium marinum* in zebrafish, an ostensibly attractive system due to genetic tractability, high-throughput and the transparency of larvae, which allows *in vivo* imaging. Determinants of Zika virus host tropism by deep mutational scanning in cerebral organoids have recently been described ^18^; this may also be a tractable system to study TBM. Attempts to develop murine models of TBM to study pathogenesis *in vivo* have been described ^19^, but have not been widely adopted, as mice incompletely replicate pathological features of TB, in particular caseous necrosis. The most commonly used animal model therefore has been the rabbit, which does recapitulate some of the pathological features of TBM ^20^. However, high dose intracisternal infection to rapidly produce neurological features also results in dissemination to other organs, which is the reverse of what is envisaged to occur in humans. Dose de-escalation studies may have a role in reproducing the early indolent phase of infection. The original description of the *Cynomolgus* model of TB, now increasingly adapted as most representative animal model of human TB, described the occurrence of meningitis ^21^, but this finding does not appear to have been exploited and may be worth revisiting.

Bacille Calmette Guérin (BCG) vaccine has shown consistently high efficacy against childhood TBM ^22^. Negative association between positivity in immune tests of *M. tuberculosis* sensitisation and prior history of vaccination ^23, 24^ encouraged the recent re-evaluation of BCG revaccination in adolescents with similar results ^25^. The inference is that BCG may have partial efficacy to prevent infection but it is unclear whether this is sufficient to explain protection against TBM. Further, it has also been shown that BCG-exposed haematopoietic stem cells generate epigenetically modified macrophages, providing protection against virulent *M. tuberculosis* infection when compared with naive macrophages ^26^. Careful correlate of protection studies of infants entering trials of novel live tuberculosis vaccines thus may have the potential to illuminate protection against TBM.

A relatively large number of studies in humans have explored the hypothesis that the inflammatory consequences of TBM are mediated by cytokine or matrix metalloproteinase (MMP) dysregulation, with consistent associations documented for tumour necrosis factor (TNF), vascular endothelial growth factor (VEGF), and MMP-9 ^1^. Similarly, a substantial number of associations between TBM and genetic polymorphism in immune response genes are also reported ^1^. Recent studies, however, have opened three further lines of enquiry. First, increased survival in microbiologically-confirmed TBM patients randomised to high dose (1000 mg) adjunctive aspirin therapy was associated with a distinct shift in the pattern of arachidonic acid metabolites ^27^, corroborating prior work by the same group that a polymorphism in the gene encoding leukotriene A4 hydrolase (LTA4H) influences both intracerebral inflammation and survival from TBM ^28^, and also associates with corticosteroid responsiveness ^29^. Second, metabolomic profiling and quantitative trait analysis demonstrated increased survival associated with decreased CSF tryptophan levels, partially under genetic influence ^30^. Third, a recent RNA sequence-based study of ventricular and lumbar CSF in children also indicated significant enrichment associated with glutamate- and GABA-mediated neuronal excitotoxicity and cerebral damage ^31^. The finding that metabolic derangement may contribute to ongoing brain damage was also suggested by a study of markers of brain injury (non-specific enolase, glial fibrillary acidic protein and S100B), which remained elevated despite a reduction of cytokine mediators in children whose outcome was poor ^32^. Taken together, these studies illuminate the power of unbiased ‘omics’ approaches using materials from the site of disease, which have great potential to better understand pathogenesis and thus illuminate new adjunctive treatments in humans.

The 2019 TBM International Research Consortium meeting in Lucknow also collated information on a significant expansion of ongoing and intended clinical trials activity in TBM which is long overdue. These trials provide an unrivalled opportunity to collect materials for *ex vivo* analysis as above and to understand interventions. They also present opportunity, where facilities exist, to non-invasively track metabolic disturbance and drug interventions by dynamic imaging (e.g. combined positron emission and computed tomographic [PET/CT], advanced magnetic resonance image [MRI] sequences, or magnetic resonance spectroscopy [MRS]). An interesting example of such an approach in the context of drug intervention was recently provided by the use of ^11^C-rifampicin PET/CT in rabbits and humans to investigate rifampicin biodistribution in TBM ^34^. Cancer imaging is more advanced in this respect, for example with the availability of a Caspase 3 tracer to detect apoptosis during treatment for cerebral malignancy ^35^. Caspase 1 and 5 mediated inflammation via inflammasome activation are implicated in TBM ^31, 36^ and the prospect to determine the anatomical distribution and potential treatment-mediated reduction of such inflammation is of interest. Knowledge gaps for TBM pathogenesis are shown in Table 2.

## Diagnosis

Lack of diagnostic suspicion and thus delay is a major contributor to poor outcome in TBM ^37^. Modalities in use include pathogen-based or host-response-based tests; and clinical algorithms, which may be supported by radiographic means where available.

Microbiological tests (microscopy, culture and nucleic amplification tests) mostly have good specificity but suboptimal sensitivity and, in the case of culture, render results too late to influence immediate management. The GeneXpert Ultra appears to provide an advance in sensitivity over the first generation of this test and, importantly, where positive, gives early indication of rifampicin resistance ^38, 39^, which presents an especially difficult management challenge. Poor sensitivity of around 22–24%, but high potentially useful ‘rule-in’ specificity, has recently been reported for lipoarabinomannan (LAM) detection in CSF or urine by means of a lateral flow assay ^40^. A second generation of this test with greater sensitivity has also recently reported and would appear to warrant prospective evaluation ^41^.

Host-response-based tests include the CSF determination of free, or antigen-specific release of, interferon-γ, adenosine deaminase, and anti-TB antibodies ^1, 42^. Diagnostic accuracy tends towards suboptimal, especially in specificity. However, multiplexed point-of-care assays to be performed either on CSF or perhaps serum with cut-offs set at useful triage levels of sensitivity may be of utility and could be evaluated further ^43^. Lumbar puncture is invasive and may fail or be impracticable in primary care and thus, blood or other body fluid-based tests applicable at the point of care may have utility. There is, for example, great interest in the application of blood-based transcriptomic assays to the diagnosis of TB and expansion of such evaluations to TBM studies may be of interest ^44^.

A substantial number of clinical algorithms have been proposed for TBM: one widely cited and in use was originally intended not for clinical diagnostic use but to help standardise research case definitions to aid comparability of studies ^45^. Prospective evaluations of such definitions against microbiological and composite clinical endpoints could be incorporated into prospective diagnostic evaluations.

A general deficiency in TBM research is that high-quality adequately-powered STARD compliant diagnostic evaluations are few ^46^. Multi-centre designs would be efficient and also potentially increase generalisability by the inclusion of important groups such as children and HIV co-infected participants, in whom diagnostic accuracy may differ. Coupled to the standardised biobanking of samples for future discovery and evaluation of novel diagnostic, this would seem an obvious priority. An interesting issue is whether such studies should be randomised to one test or another to best-reflect the likely clinical application that only one test would be performed. Concentration techniques applied to CSF consistently improve microbiological yield: in clinical practice large volumes of CSF tend not to be drawn for TB tests. However, the efficient alternative is to divide the same CSF sample for different diagnostic tests. In the context of a prospective well-designed research evaluation, a greater volume than may be considered routine (15–20 ml in adults) is regarded as safe and feasible. Knowledge gaps for the diagnosis of TBM are shown in Table 3.

## Antimicrobial therapy

One intuitive way to improve outcome of TBM is by intensifying antimicrobial treatment ^47^. Transporters, like P-glycoprotein (P-gp), actively pump substances out of the brain and into the blood or CSF, to protect the CNS from the free entry of potentially neurotoxic substances ^48^. These pumps also lower penetration of some first-line TB drugs, especially rifampicin and ethambutol. Also, it is largely unknown how the anatomical distribution, quantity and metabolic status of *M. tuberculosis* bacilli in TBM affect the killing effect of specific anti-tuberculous drugs, and if antimicrobial killing may induce (damaging) host inflammatory responses in TBM.

There are different strategies to optimize TBM treatment. Prolonging treatment is unlikely to help; in a meta-analysis, the risk of relapse was extremely low (~0.8%), and virtually identical in patients who received <6 months versus >6 months of therapy ^49^. A more promising strategy is to increase drug exposures in the CNS by increasing doses of poorly penetrating drugs. In a phase II clinical trial in Indonesia, rifampicin administered intravenously (600 mg, or 13 mg/kg iv) for two weeks resulted in a three-fold higher exposure to rifampicin in plasma and CSF than 450mg (~10 mg/kg) given orally, and a 50% lower mortality ^50^. In contrast, a large trial involving 817 TBM patients in Vietnam did not show a survival benefit of an intensified regimen with 15 (rather than 10) mg/kg of oral rifampicin plus levofloxacin versus standard of care for eight weeks ^51^, except for patients with isoniazid-mono-resistant TBM ^52^. The lack of an effect in that study might be explained by the relatively modest dose increase of rifampicin, as modelling of >1100 pharmacokinetic measurements and survival data from 133 TBM patients in Indonesia suggest that rifampicin exposure strongly predicts survival and that the optimal oral dose is likely to be 35 mg/kg or higher ^53^. These findings were corroborated in a study that examined rifampicin cerebral distribution in a rabbit model of TBM ^34^. This dose is used in a phase 3 randomised controlled trial (RCT) to be conducted in Uganda, South Africa and Indonesia (ISRCTN: 15668391), and other RCTs that are planned.

Although isoniazid has excellent CSF penetration ^54^, its dose may also be important. Isoniazid is metabolized by the genetically polymorphic N-acetyltransferase 2 (NAT2), and rapid acetylators have lower isoniazid exposure in plasma and CSF ^55^, and might benefit from higher doses.

Fluoroquinolones have also been examined, but no effect was seen, both in Indonesia ^50^ and Vietnam ^51^. Other second-line or new drugs that may provide meaningful benefit include cycloserine or ethionamide (though these drugs carry high risk of toxicity) ^56^, linezolid, and possibly the nitroimidazoles (e.g. delamanid or pretomanid) ^57^. It is hoped that new compounds in the pipeline will have characteristics associated with high probability of achieving effective concentrations in the brain and CSF.

Besides higher drug doses or new drugs, modification of drug delivery (e.g. by liposome or nanoparticle-mediated permeation), or inhibition of efflux pumps might also increase drug exposure and effect ^48, 58, 59^. Knowledge gaps for antimicrobial drug therapy in TBM are shown in Table 4.

## Host directed therapies

The importance of intra-cerebral inflammation to the complications and outcome of TBM has been appreciated ever since it became a treatable disease in the late 1940s. Streptomycin, the first anti-tuberculosis drug to be trialled in humans, converted TBM from a universally fatal disease to one in which around 30–40% survived ^60^. Contemporaneous with this breakthrough was the notion that outcomes might be further improved if inflammation was controlled whilst antibiotics killed the bacteria.

Corticosteroids were the first host directed therapy to be employed in TBM treatment, with trials published in the early 1950s suggesting they caused faster resolution of symptoms and CSF inflammatory indices ^61^. However, it took 50 more years to acquire the definitive data from randomised controlled trials that showed adjunctive corticosteroids reduced deaths from TBM ^2^.

Corticosteroids for TBM remain the only host directed therapy for any form of tuberculosis with high-quality randomised controlled trial evidence of benefit. There are, however, many reasons to be dissatisfied with them as therapeutic agents. First, they reduce deaths, but do not reduce disability. Second, their benefit appears heterogenous; some patients respond rapidly, with marked improvements in clinical and radiological parameters, whilst others do not respond at all. Third, the mechanism by which they reduce deaths is unknown; it does not, for example, appear to associate with a measurable anti-inflammatory effect ^62^. Finally, corticosteroids have a panoply of well-recognised side effects, that can limit their use.

The limitations of corticosteroids as host directed therapy have stimulated various avenues of investigation, all of which represent current research priorities. There is much interest in trying to understand the underlying mechanisms of their therapeutic heterogeneity and how they reduce the risk of death in some people with TBM but not others. The objective of these investigations is to identify the relevant molecules or pathways, which may be targeted by more effective and less toxic drugs.

Clinical necessity in those with corticosteroid-refractory disease has driven the use and study of other available anti-inflammatory drugs. Their selection has mostly been predicated on the hypothesis that tumour necrosis factor (TNF) is a critical cytokine in TBM pathogenesis and its inhibition may improve clinical outcomes. To this end, thalidomide and the anti-TNF biological drugs have all been studied, but only in individual reports or small case series ^1^. Adequately powered clinical trials have yet to be instigated with these agents.

There is growing interest in the use of aspirin as a host directed therapy for TBM. Brain infarcts are an important cause of disability following TBM and are not reduced by corticosteroids. Thus, the hypothesis that aspirin might prevent infarcts is attractive, but to date has only been subject to three relatively small phase 2 clinical trials ^27, 63, 64^. The most recent trial compared low dose (81mg/day) with higher dose (1000mg/day) aspirin for the first 60 days treatment of 120 adults, complementing clinical response assessments with serial brain imaging and the measurement of lipid inflammatory mediators in the CSF ^27^. The results suggested higher dose aspirin may reduce infarcts and deaths, possibly through the up-regulation of lipid mediators that stimulate inflammation resolution. Large phase 3 trials of adjunctive aspirin are now a priority.

Aside from research driven by the benefit and limitations of corticosteroids, and the exploration of other already available anti-inflammatory agents, the priority is to better understand the pathophysiology of TBM. This approach may expose novel pathways and molecules amenable to drugs and could lead to innovative new host directed therapies. It will likely require coordinated investigations that link cellular and animal disease models with careful clinical studies characterising molecular determinants of outcome. The application of ‘omics’ technologies and analyses may assist in the search for new drug targets. Knowledge gaps for host directed therapy in TBM are shown in Table 5.

## Critical care

Critical care, or acute supportive care, is important because neurological disability and mortality commonly occurs early after presentation. TBM pathology initiates secondary mechanisms such as cerebral ischemia, increased intracranial pressure (ICP), excitotoxicity, and other metabolic derangements ^30, 31^, all of which may interact in a negative downward spiral. Several factors limit our current ability to detect and respond to such derangements, including lack of laboratory and bedside tools to study the disease processes, limited capacity for advanced brain imaging, and few neuromonitoring strategies, compounded by the general lack of resources at sites where TBM is most common. Lack of clinicians trained in critical care and surgical care at these sites further limits options for clinical intervention and research. Late diagnosis may lead to consequences of cerebral pathology too advanced for therapeutic intervention to make a significant difference. Key aspects of initial clinical treatment of TBM are not standardised and practice at different centres is widely divergent. For example, even though hydrocephalus is common in TBM and is the leading cause of increased ICP, there is no consensus on optimal care, including the evaluation of increased ICP, the role of the level of CSF block (communicating versus non-communicating hydrocephalus), indications for medical therapy, criteria for surgical intervention, or the choice of surgical technique ^65^.

Although there are small single centre randomised trials of surgical technique, selection criteria vary, and the studies have major limitations. Similarly, hyponatremia is common and associates with poor outcome. Hyponatraemia may be caused by cerebral salt wasting, or the syndrome of inappropriate ADH secretion, or both, and distinguishing between them is difficult. This is important because optimal treatment depends on the diagnosis, and inappropriate care may lead to harm. Protocols for safe and effective amelioration of hyponatremia are needed ^3^. Supportive systemic care, including haemodynamic and respiratory support, are important in a general sense, but whether more aggressive interventions, such as blood pressure augmentation and optimization of systemic and cerebral oxygenation, can limit cerebral ischemia remains unknown.

Advanced monitoring of the brain to detect derangements in perfusion and metabolism has been increasingly employed for other acute neurological conditions, but there is little experience of these in TBM and the costs are substantial ^66, 67^. Non-invasive means to detect increased ICP or impaired brain perfusion have been used in several conditions with variable success and remain under investigation ^68– 71^. Finally, early identification of patients at high risk of neurological deterioration, based on clinical criteria, radiological features, and/or biomarkers, could help direct more aggressive care before permanent infarction occurs. Knowledge gaps for the critical care of individuals with TBM are shown in Table 6.

## The cascade of care for tuberculous meningitis

Mortality of TBM varies between settings and this may be due to specific variation in the availability and quality of health care services, both prior to, during and after hospitalization. It was recently estimated that 50% of TB deaths result from poor-quality care ^72^. For TBM it might be even worse, as it requires advanced diagnostics and specialized care, which are often either absent or suboptimal in low-resource settings.

Care for TB patients comprises a cascade of essential steps, with each step unable individually to guarantee a good outcome. It starts with the number of TB patients, followed by the number of patients that accesses TB services for testing, the number diagnosed, the number started on treatment, and finally the number successfully completing treatment ^73^. For TBM, the cascade should probably be adjusted to include additional steps such as ‘discharged alive’ or ‘retained to care after discharge’ and ‘those completing treatment without significant disability’ ^33^. Some studies have also measured the time between steps ^74^. This will be important for TBM, which can be rapidly progressive if no diagnosis is made or treatment started.

Patient pathway studies, which asses the alignment of health systems’ infrastructure (e.g. diagnostic, referral and treatment capacity) with patients’ care-seeking behavior, could help identify factors that account for losses or delays across a cascade of care for TBM ^75^. Many of the possible gaps in diagnosis and treatment of TBM are related to health systems factors, such as the availability of the right facilities or workforce, health information, guidelines, drugs, financing, and organization of the healthy system. A health needs assessment framework measures indicators of performance across system parameters and quantifies the gaps in care against an ‘ideal’ system, and then considers, using pre-determined criteria, different options to fill each gap. Such public health frameworks have previously been used to identify gaps between current and ideal practice for management of child-case TB contacts ^76^, and interventions based on this framework are now tested in a multi-country cluster-randomized clinical trial ^77^. Establishing the cascade of care for TBM, conducting a patient pathway analysis, and further study of health systems factors could thus help identify priority areas for further action to improve care and outcomes for TBM patients. Some of the knowledge gaps are presented in Table 7, and the theoretical framework is presented in more detail elsewhere in this supplement ^33^.

## Conclusions

TBM is the most severe form of TB and contributes disproportionately to TB mortality and morbidity. In order to improve the care of patients with this devastating condition we need to have a better understanding of the disease epidemiology and pathogenesis, as well as improved diagnostic tests and treatment strategies. These then need to be incorporated into the health systems in which care is delivered. Many questions remain unanswered and much research is required. However, the process of defining these questions and deciding on areas of priority allows researchers to focus and coordinate their efforts and allows funders a clearer idea of how and where to spend their money.

## Data availability

No data are associated with this article.

## References

[1] Wilkinson RJ Rohlwink U Misra UK: Tuberculous meningitis. *Nat Rev Neurol.* 2017;13(10):581–598. 10.1038/nrneurol.2017.120 28884751

[2] Prasad K Singh MB Ryan H: Corticosteroids for managing tuberculous meningitis. *Cochrane Database Syst Rev.* 2016;4:CD002244. 10.1002/14651858.CD002244.pub4 27121755 PMC4916936

[3] Donovan J Figaji A Imran D: The neurocritical care of tuberculous meningitis. *Lancet Neurol.* 2019;18(8):771–783. 10.1016/S1474-4422(19)30154-1 31109897

[4] Gupta RK Lucas SB Fielding KL: Prevalence of tuberculosis in post-mortem studies of HIV-infected adults and children in resource-limited settings: a systematic review and meta-analysis. *AIDS.* 2015;29(15):1987–2002. 10.1097/QAD.0000000000000802 26266773 PMC4568896

[5] Wolzak NK Cooke ML Orth H: The changing profile of pediatric meningitis at a referral centre in Cape Town, South Africa. *J Trop Pediatr.* 2012;58(6):491–495. 10.1093/tropej/fms031 22791086

[6] World Health Organization, Geneva, Switzerland: Global Tuberculosis Report.(accessed 10 December 2018).2018 Reference Source

[7] World Health Organization, Geneva, Switzerland: Definitions and reporting framework for tuberculosis. 2013; revision, updated December 2014. (accessed 4 October 2019). Reference Source

[8] Ducomble T Tolksdorf K Karagiannis I: The burden of extrapulmonary and meningitis tuberculosis: an investigation of national surveillance data, Germany, 2002 to 2009. *Euro Surveill.* 2013;18(12):pii: 20436. 23557944

[9] Seddon JA Hesseling AC Marais BJ: The evolving epidemic of drug-resistant tuberculosis among children in Cape Town, South Africa. *Int J Tuberc Lung Dis.* 2012;16(7):928–933. 10.5588/ijtld.11.0679 22583610

[10] Lincoln EM: Tuberculous meningitis in children; with special reference to serous meningitis; tuberculous meningitis. *Am Rev Tuberc.* 1947;56(2):75–94. 20264373 10.1164/art.1947.56.2.75

[11] Chiang SS Khan FA Milstein MB: Treatment outcomes of childhood tuberculous meningitis: a systematic review and meta-analysis. *Lancet Infect Dis.* 2014;14(10):947–957. 10.1016/S1473-3099(14)70852-7 25108337

[12] Thwaites GE Duc Bang N Huy Dung N: The influence of HIV infection on clinical presentation, response to treatment, and outcome in adults with Tuberculous meningitis. *J Infect Dis.* 2005;192(12):2134–2141. 10.1086/498220 16288379

[13] Cecchini D Ambrosioni J Brezzo C: Tuberculous meningitis in HIV-infected and non-infected patients: comparison of cerebrospinal fluid findings. *Int J Tuberc Lung Dis.* 2009;13(2):269–271. 19146759

[14] Davis AG Rohlwink UK Proust A: The pathogenesis of tuberculous meningitis. *J Leukoc Biol.* 2019;105(2):267–280. 10.1002/JLB.MR0318-102R 30645042 PMC6355360

[15] Rich AR: The pathogenesis of tuberculosis.Springfield, IL: C.C. Thomas.1946.

[16] Donald PR Schaaf HS Schoeman JF: Tuberculous meningitis and miliary tuberculosis: the Rich focus revisited. *J Infect.* 2005;50(3):193–195. 10.1016/j.jinf.2004.02.010 15780412

[17] van Leeuwen LM Boot M Kuijl C: Mycobacteria employ two different mechanisms to cross the blood-brain barrier. *Cell Microbiol.* 2018;20(9):e12858. 10.1111/cmi.12858 29749044 PMC6175424

[18] Setoh YX Amarilla AA Peng NYG: Determinants of Zika virus host tropism uncovered by deep mutational scanning. *Nat Microbiol.* 2019;4(5):876–887. 10.1038/s41564-019-0399-4 30886357 PMC6667831

[19] Hernandez Pando R Aguilar D Cohen I: Specific bacterial genotypes of *Mycobacterium tuberculosis* cause extensive dissemination and brain infection in an experimental model. *Tuberculosis (Edinb).* 2010;90(4):268–277. 10.1016/j.tube.2010.05.002 20580613

[20] Tsenova L Sokol K Freedman VH: A combination of thalidomide plus antibiotics protects rabbits from mycobacterial meningitis-associated death. *J Infect Dis.* 1998;177(6):1563–1572. 10.1086/515327 9607834

[21] Walsh GP Tan EV dela Cruz EC: The Philippine cynomolgus monkey ( *Macaca fasicularis*) provides a new nonhuman primate model of tuberculosis that resembles human disease. *Nat Med.* 1996;2(4):430–436. 10.1038/nm0496-430 8597953

[22] Trunz BB Fine P Dye C: Effect of BCG vaccination on childhood tuberculous meningitis and miliary tuberculosis worldwide: a meta-analysis and assessment of cost-effectiveness. *Lancet.* 2006;367(9517):1173–1180. 10.1016/S0140-6736(06)68507-3 16616560

[23] Soysal A Millington KA Bakir M: Effect of BCG vaccination on risk of *Mycobacterium tuberculosis* infection in children with household tuberculosis contact: a prospective community-based study. *Lancet.* 2005;366(9495):1443–1451. 10.1016/S0140-6736(05)67534-4 16243089

[24] Oni T Gideon HP Bangani N: Smoking, BCG and Employment and the Risk of Tuberculosis Infection in HIV-Infected Persons in South Africa. *PLoS One.* 2012;7(10):e47072. 10.1371/journal.pone.0047072 23056584 PMC3467259

[25] Nemes E Geldenhuys H Rozot V: Prevention of *M. tuberculosis* Infection with H4:IC31 Vaccine or BCG Revaccination. *N Engl J Med.* 2018;379(2):138–149. 10.1056/NEJMoa1714021 29996082 PMC5937161

[26] Kaufmann E Sanz J Dunn JL: BCG Educates Hematopoietic Stem Cells to Generate Protective Innate Immunity against Tuberculosis. *Cell.* 2018;172(1–2):176–190.e19. 10.1016/j.cell.2017.12.031 29328912

[27] Mai NT Dobbs N Phu NH: A randomised double blind placebo controlled phase 2 trial of adjunctive aspirin for tuberculous meningitis in HIV-uninfected adults. *eLife.* 2018;7: pii:e33478. 10.7554/eLife.33478 29482717 PMC5862527

[28] Thuong NTT Heemskerk D Tram TTB: Leukotriene A4 Hydrolase Genotype and HIV Infection Influence Intracerebral Inflammation and Survival From Tuberculous Meningitis. *J Infect Dis.* 2017;215(7):1020–1028. 10.1093/infdis/jix050 28419368 PMC5426373

[29] Tobin DM Roca FJ Oh SF: Host genotype-specific therapies can optimize the inflammatory response to mycobacterial infections. *Cell.* 2012;148(3):434–446. 10.1016/j.cell.2011.12.023 22304914 PMC3433720

[30] van Laarhoven A Dian S Aguirre-Gamboa R: Cerebral tryptophan metabolism and outcome of tuberculous meningitis: an observational cohort study. *Lancet Infect Dis.* 2018;18(5):526–535. 10.1016/S1473-3099(18)30053-7 29395996

[31] Rohlwink UK Figaji A Wilkinson KA: Tuberculous meningitis in children is characterized by compartmentalized immune responses and neural excitotoxicity. *Nat Commun.* 2019;10(1):3767. 10.1038/s41467-019-11783-9 31434901 PMC6704154

[32] Rohlwink UK Mauff K Wilkinson KA: Biomarkers of Cerebral Injury and Inflammation in Pediatric Tuberculous Meningitis. *Clin Infect Dis.* 2017;65(8):1298–1307. 10.1093/cid/cix540 28605426 PMC5815568

[33] Imran D Hill PC McKnight J: Establishing the cascade of care for patients with tuberculous meningitis [version 1; peer review: awaiting peer review]. *Wellcome Open Res.* 2019;4:177 10.12688/wellcomeopenres.15515.1 32118119 PMC7008603

[34] Tucker EW Guglieri-Lopez B Ordonez AA: Noninvasive ^11^C-rifampin positron emission tomography reveals drug biodistribution in tuberculous meningitis. *Sci Transl Med.* 2018;10(470): pii:eaau0965. 10.1126/scitranslmed.aau0965 30518610 PMC6360528

[35] Nguyen QD Challapalli A Smith G: Imaging apoptosis with positron emission tomography: 'bench to bedside' development of the caspase-3/7 specific radiotracer [ ^18^F]ICMT-11. *Eur J Cancer.* 2012;48(4):432–440. 10.1016/j.ejca.2011.11.033 22226480

[36] Marais S Lai RPJ Wilkinson KA: Inflammasome Activation Underlying Central Nervous System Deterioration in HIV-Associated Tuberculosis. *J Infect Dis.* 2017;215(5):677–686. 10.1093/infdis/jiw561 27932622 PMC5388298

[37] Thwaites GE van Toorn R Schoeman J: Tuberculous meningitis: more questions, still too few answers. *Lancet Neurol.* 2013;12(10):999–1010. 10.1016/S1474-4422(13)70168-6 23972913

[38] Bahr NC Nuwagira E Evans EE: Diagnostic accuracy of Xpert MTB/RIF Ultra for tuberculous meningitis in HIV-infected adults: a prospective cohort study. *Lancet Infect Dis.* 2018;18(1):68–75. 10.1016/S1473-3099(17)30474-7 28919338 PMC5739874

[39] Wang G Wang S Jiang G: Xpert MTB/RIF Ultra improved the diagnosis of paucibacillary tuberculosis: A prospective cohort study. *J Infect.* 2019;78(4):311–316. 10.1016/j.jinf.2019.02.010 30796951

[40] Siddiqi OK Birbeck GL Ghebremichael M: Prospective Cohort Study on Performance of Cerebrospinal Fluid (CSF) Xpert MTB/RIF, CSF Lipoarabinomannan (LAM) Lateral Flow Assay (LFA), and Urine LAM LFA for Diagnosis of Tuberculous Meningitis in Zambia. *J Clin Microbiol.* 2019;57(8): pii:e00652-19. 10.1128/JCM.00652-19 31189584 PMC6663887

[41] Broger T Sossen B du Toit E: Novel lipoarabinomannan point-of-care tuberculosis test for people with HIV: a diagnostic accuracy study. *Lancet Infect Dis.* 2019;19(8):852–861. 10.1016/S1473-3099(19)30001-5 31155318 PMC6656794

[42] Huang TY Zhang XX Wu QL: Antibody detection tests for early diagnosis in tuberculous meningitis. *Int J Infect Dis.* 2016;48:64–69. 10.1016/j.ijid.2016.05.007 27173078

[43] Manyelo CM Solomons RS Snyders CI: Application of Cerebrospinal Fluid Host Protein Biosignatures in the Diagnosis of Tuberculous Meningitis in Children from a High Burden Setting. *Mediators Inflamm.* 2019;2019:7582948. 10.1155/2019/7582948 31148946 PMC6501148

[44] Singhania A Wilkinson RJ Rodrigue M: The value of transcriptomics in advancing knowledge of the immune response and diagnosis in tuberculosis. *Nat Immunol.* 2018;19(11):1159–1168. 10.1038/s41590-018-0225-9 30333612 PMC6554194

[45] Marais S Thwaites G Schoeman JF: Tuberculous meningitis: a uniform case definition for use in clinical research. *Lancet Infect Dis.* 2010;10(11):803–812. 10.1016/S1473-3099(10)70138-9 20822958

[46] Heemskerk AD Donovan J Thu DDA: Improving the microbiological diagnosis of tuberculous meningitis: A prospective, international, multicentre comparison of conventional and modified Ziehl-Neelsen stain, GeneXpert, and culture of cerebrospinal fluid. *J Infect.* 2018;77(6):509–515. 10.1016/j.jinf.2018.09.003 30217659 PMC6293313

[47] Cresswell FV Te Brake L Atherton R: Intensified antibiotic treatment of tuberculosis meningitis. *Expert Rev Clin Pharmacol.* 2019;12(3):267–288. 10.1080/17512433.2019.1552831 30474434

[48] Te Brake LHM de Knegt GJ de Steenwinkel JE: The Role of Efflux Pumps in Tuberculosis Treatment and Their Promise as a Target in Drug Development: Unraveling the Black Box. *Annu Rev Pharmacol Toxicol.* 2018;58:271–291. 10.1146/annurev-pharmtox-010617-052438 28715978

[49] Jullien S Ryan H Modi M: Six months therapy for tuberculous meningitis. *Cochrane Database Syst Rev.* 2016;9:CD012091. 10.1002/14651858.CD012091.pub2 27581996 PMC5018659

[50] Ruslami R Ganiem AR Dian S: Intensified regimen containing rifampicin and moxifloxacin for tuberculous meningitis: an open-label, randomised controlled phase 2 trial. *Lancet Infect Dis.* 2013;13(1):27–35. 10.1016/S1473-3099(12)70264-5 23103177

[51] Heemskerk AD Bang ND Mai NT: Intensified Antituberculosis Therapy in Adults with Tuberculous Meningitis. *N Engl J Med.* 2016;374(2):124–134. 10.1056/NEJMoa1507062 26760084

[52] Heemskerk AD Nguyen MTH Dang HTM: Clinical Outcomes of Patients With Drug-Resistant Tuberculous Meningitis Treated With an Intensified Antituberculosis Regimen. *Clin Infect Dis.* 2017;65(1):20–28. 10.1093/cid/cix230 28472255 PMC5850451

[53] Svensson EM Dian S Te Brake L: Model-based meta-analysis of rifampicin exposure and mortality in Indonesian tuberculosis meningitis trials. *Clin Infect Dis.* 2019; pii: ciz1071. 10.1093/cid/ciz1071 31665299 PMC7643733

[54] Donald PR: Cerebrospinal fluid concentrations of antituberculosis agents in adults and children. *Tuberculosis (Edinb).* 2010;90(5):279–292. 10.1016/j.tube.2010.07.002 20709598

[55] Yunivita V Ruslami R Dian S: Pharmacokinetics of isoniazid and the effect of acetylator status in Indonesian tuberculous meningitis patients. *10th International Workshop on Clinical Pharmacology of TB drugs;*October 16, 2017; Atlanta, USA.2017.

[56] Alvarez-Uria G Midde M Pakam R: Initial Antituberculous Regimen with Better Drug Penetration into Cerebrospinal Fluid Reduces Mortality in HIV Infected Patients with Tuberculous Meningitis: Data from an HIV Observational Cohort Study. *Tuberc Res Treat.* 2013;2013:242604. 10.1155/2013/242604 23997952 PMC3753756

[57] Tucker EW Pieterse L Zimmerman MD: Delamanid Central Nervous System Pharmacokinetics in Tuberculous Meningitis in Rabbits and Humans. *Antimicrob Agents Chemother.* 2019;63(10): pii: e00913-19. 10.1128/AAC.00913-19 31383662 PMC6761520

[58] Begley DJ: Delivery of therapeutic agents to the central nervous system: the problems and the possibilities. *Pharmacol Ther.* 2004;104(1):29–45. 10.1016/j.pharmthera.2004.08.001 15500907

[59] Mustafa S Pai RS Singh G: Nanocarrier-based interventions for the management of MDR/XDR-TB. *J Drug Target.* 2015;23(4):287–304. 10.3109/1061186X.2015.1009076 25766078

[60] Cairns H Duthie ES: Intrathecal streptomycin in meningitis; clinical trial in tuberculous, coliform, and other infections. *Lancet.* 1946;2(6414):153–155. 10.1016/s0140-6736(46)90728-3 20995251

[61] Shane SJ Riley C: Tuberculous meningitis: combined therapy with cortisone and antimicrobial agents. *N Engl J Med.* 1953;249(21):829–834. 10.1056/NEJM195311192492101 13111361

[62] Simmons CP Thwaites GE Quyen NT: The clinical benefit of adjunctive dexamethasone in tuberculous meningitis is not associated with measurable attenuation of peripheral or local immune responses. *J Immunol.* 2005;175(1):579–590. 10.4049/jimmunol.175.1.579 15972695

[63] Schoeman JF Janse van Rensburg A Laubscher JA: The role of aspirin in childhood tuberculous meningitis. *J Child Neurol.* 2011;26(8):956–962. 10.1177/0883073811398132 21628697

[64] Misra UK Kalita J Nair PP: Role of aspirin in tuberculous meningitis: a randomized open label placebo controlled trial. *J Neurol Sci.* 2010;293(1–2):12–17. 10.1016/j.jns.2010.03.025 20421121

[65] Figaji AA Fieggen AG: The neurosurgical and acute care management of tuberculous meningitis: evidence and current practice. *Tuberculosis (Edinb).* 2010;90(6):393–400. 10.1016/j.tube.2010.09.005 20970381

[66] Le Roux P Menon DK Citerio G: Consensus summary statement of the International Multidisciplinary Consensus Conference on Multimodality Monitoring in Neurocritical Care: a statement for healthcare professionals from the Neurocritical Care Society and the European Society of Intensive Care Medicine. *Neurocrit Care.* 2014;21(Suppl 2):S1–26. 10.1007/s12028-014-0041-5 25208678 PMC10596301

[67] Figaji AA Sandler SI Fieggen AG: Continuous monitoring and intervention for cerebral ischemia in tuberculous meningitis. *Pediatr Crit Care Med.* 2008;9(4):e25–30. 10.1097/PCC.0b013e318172e8b7 18843248

[68] Tai MS Sharma VK: Role of Transcranial Doppler in the Evaluation of Vasculopathy in Tuberculous Meningitis. *PLoS One.* 2016;11(10):e0164266. 10.1371/journal.pone.0164266 27723828 PMC5056701

[69] van Toorn R Schaaf HS Solomons R: The value of transcranial Doppler imaging in children with tuberculous meningitis. *Childs Nerv Syst.* 2014;30(10):1711–6. 10.1007/s00381-014-2435-2 24828794

[70] Sangani SV Parikh S: Can sonographic measurement of optic nerve sheath diameter be used to detect raised intracranial pressure in patients with tuberculous meningitis? A prospective observational study. *Indian J Radiol Imaging.* 2015;25(2):173–176. 10.4103/0971-3026.155869 25969641 PMC4419427

[71] Nabeta HW Bahr NC Rhein J: Accuracy of noninvasive intraocular pressure or optic nerve sheath diameter measurements for predicting elevated intracranial pressure in cryptococcal meningitis. *Open Forum Infect Dis.* 2014;1(3):ofu093. 10.1093/ofid/ofu093 25734161 PMC4324219

[72] Reid MJ Arinaminpathy N Bloom A: Building a tuberculosis-free world: The *Lancet* Commission on tuberculosis. *Lancet.* 2019;393(10178):1331–1384. 10.1016/S0140-6736(19)30024-8 30904263

[73] Naidoo P Theron G Rangaka MX: The South African Tuberculosis Care Cascade: Estimated Losses and Methodological Challenges. *J Infect Dis.* 2017;216(suppl_7):S702–S713. 10.1093/infdis/jix335 29117342 PMC5853316

[74] Soeroto AY Lestari BW Santoso P: Evaluation of Xpert *MTB-RIF* guided diagnosis and treatment of rifampicin-resistant tuberculosis in Indonesia: A retrospective cohort study. *PLoS One.* 2019;14(2):e0213017. 10.1371/journal.pone.0213017 30818352 PMC6394995

[75] Hanson CL Osberg M Brown J: Conducting Patient-Pathway Analysis to Inform Programming of Tuberculosis Services: Methods. *J Infect Dis.* 2017;216(suppl_7):S679–S685. 10.1093/infdis/jix387 29117350 PMC5853893

[76] Rutherford ME Ruslami R Anselmo M: Management of children exposed to *Mycobacterium tuberculosis*: a public health evaluation in West Java, Indonesia. *Bull World Health Organ.* 2013;91(12):932–941A. 10.2471/BLT.13.118414 24347732 PMC3845271

[77] Oxlade O Trajman A Benedetti A: Enhancing the public health impact of latent tuberculosis infection diagnosis and treatment (ACT4): protocol for a cluster randomised trial. *BMJ Open.* 2019;9(3):e025831. 10.1136/bmjopen-2018-025831 30898826 PMC6527985

