# Supplementary material for: Knowledge gaps and research priorities in tuberculous meningitis
Source: Wellcome Open Res. 2019 Nov 28;4:188. [Version 1] doi: 10.12688/wellcomeopenres.15573.1 (PMC7014926; doi:10.12688/wellcomeopenres.15573.1)
Supplement: Peer review history

# Peer review: Reviewer response for version 1

**DOI:** 10.21956/wellcomeopenres.17054.r37818

**Published:** 2020-02-11

**Authors:** Jamie Rylance

**Affiliations:** 1 Liverpool School of Tropical Medicine, Liverpool, UK

The letter describes a consortium meeting comprising experts from wide geographic scope and areas of research. It is an accessible summary of the symposium.

 It would be helpful to give more detail about the processes taken to define knowledge gaps and prioritise the research foci, although perhaps not required in a letter. Specifically:

1. How were the research updates produced? Incomplete or partial summaries would have the potential to skew subsequent discussion - which methods were employed to mitigate against this?
2. Are the knowledge gaps presented in the latter an exhaustive list of those identified during discussions? If not, what selection processes took place?
3. Table 8 priorities were derived from the themed discussions on knowledge gaps - was the prioritisation formalised, and if so, what methods were used?

Minor points:

1. Some tables refer to "correlate of protection" studies (notated 'C'). Is this intended to convey a case-control approach? If not, how does this category differ from those described as "observational studies" (notated 'D').
2. "and organisation of the healthy system" needs correction.

I confirm that I have read this submission and believe that I have an appropriate level of expertise to confirm that it is of an acceptable scientific standard.

# Peer review: Reviewer response for version 1

**DOI:** 10.21956/wellcomeopenres.17054.r37190

**Published:** 2020-01-24

**Authors:** Christopher Vinnard

**Affiliations:** 1 Public Health Research Institute, Rutgers, The State University of New Jersey, Newark, NJ, USA

Dr. Seddon *et al.* provide a comprehensive summary of tuberculous meningitis research priorities, arising from discussions during the 3 ^rd^ TBM International Research Consortium that met in March 2019. The authors have summarized knowledge gaps and research priorities related to tuberculous meningitis epidemiology, pathogenesis, diagnostics, antimicrobial therapies, host-directed therapies, critical care support, and cascade of care. Under each area, specific questions are presented alongside potential study designs (both pre-clinical and clinical). Overall, the manuscript is clearly written, concise, and provides a valuable roadmap towards synergizing diverse research efforts towards the common purpose of improving patient outcomes for tuberculous meningitis. I have only a few minor comments to consider.

 The value of biomarkers of treatment response is included in several areas (epidemiology, therapeutics, critical care). I would add the tremendous potential value of an early treatment response biomarker in supporting greater efficiency in the design of clinical trials of novel therapies, whether pathogen- or host-directed, including the potential for adapative clinical trial designs.

 Second, the role of clinical algorithms in the diagnosis of tuberculous meningitis is highlighted as a knowledge gap, stressing the importance of including key sub-groups to support generalizability. It might be worthwhile to stress that a multicenter design, which includes both derivation and validation cohorts, is essential given the role of patient genetic factors in pathogenesis and treatment response. The geographic variability in the local epidemiology of central nervous system diseases, which compete with tuberculous meningitis on the differential diagnosis, can also be addressed by appropriate multicenter designs.

 Finally, although perhaps less pressing than the acute issues surrounding timely diagnosis and initiation of effective antimicrobial therapy, there continues to be uncertainty regarding the appropriate duration of therapy for tuberculous meningitis, with regards to the risk of relapse. This is a particular challenge with rifampin-resistant disease. For example, what is the role of serial imaging (where available) to guide clinical decision-making for cessation of treatment. Although these questions appear to be the focus of a separate review stemming from the Consortium, it could be worthwhile to highlight this question as well in the broader overview.

 In summary, Dr. Seddon *et al.* have developed an essential framework for the tuberculous meningitis research community, which should be of great value to investigators, public health authorities, and funding agencies.

I confirm that I have read this submission and believe that I have an appropriate level of expertise to confirm that it is of an acceptable scientific standard.
